# Redox equilibrium of serum apolipoprotein E3: a buffering effect of disulfide-linked complexes against oxidative stress on apolipoprotein E3–containing lipoproteins

**DOI:** 10.1042/BSR20190184

**Published:** 2019-04-30

**Authors:** Kazuyoshi Yamauchi, Shio Iwasaki, Yasushi Kawakami

**Affiliations:** Department of Laboratory Medicine, Faculty of Medicine, University of Tsukuba, Japan

**Keywords:** Alzheimer’s disease, apoE-AII complex, Band-shift assay, Cysteine thiol, HDL, Oxidation

## Abstract

Reversible redox modification of cysteine thiols is crucial for protecting proteins from irreversible detrimental change. However, the physiological significance of the redox modification of apolipoprotein (apo) E is unclear. Here, we hypothesized that the disulfide-linked complexes of apoE3 corresponding to the representative reversible-modified apoE3 play a protective role against oxidative stress. The effects of disulfide bond formation on oxidative stress on apoE3 were evaluated with a band-shift assay. Maleimide-labeled apoE3 and unlabeled apoE3 were defined as the reduced (r)-apoE3 and non-reduced (nr)-apoE3 forms, respectively. Hydrogen peroxide-induced oxidation decreased for reduced-form apoE (r-apoE3) but increased for nr-apoE3. Induction of apoE3-AII complex formation with excess of apoAII markedly suppressed the oxidative stress-induced increase in nr-apoE3 (*P*<0.001) and enhanced homodimer formation. The apoE3-AII complex was more dominant in high-density lipoprotein (HDL) than in very low-density lipoprotein. Under oxidative stress, HDL showed a significant decrease, rather than an increase, in nr-apoE3 levels with a concomitant significant increase in apoE3-AII levels (*P*<0.005). This finding suggests that the majority of nr-apoE3 in HDL exists in a reversible oxidized form. The apoE3-AII complex, formed from the reversible oxidized apoE3, is beneficial for maintaining the redox equilibrium of apoE3 by preventing the modification of apoE3 to its irreversible oxidized form. The apoE3-AII complex may be possibly implicated in the pathophysiology of various apoE-related diseases.

## Introduction

Apolipoprotein E (apoE), a 35-kDa glycoprotein comprising 299 amino acids residues, is primarily synthesized in the liver and plays a pivotal role in lipid transport and metabolism [1]. In addition, it is expressed in the brain and is involved not only in cerebral lipid metabolism, but also in the growth and repair of the central nervous system [2]. Human apoE has three major isoforms—apoE2, apoE3, and apoE4—that are the products of three independent alleles at a single locus [1]. The structural variances in these isoforms have led to functional changes and pathological consequences [2]. In fact, apoE has been shown to play a role in the development of various diseases [3]. In particular, apoE4 is a major causative factor of sporadic late-onset Alzheimer’s disease (AD) and its frequency is markedly increased in AD [4]. On the other hand, in another study, apoE4 was not present in approximately 30% of the patients with AD [5]. Although the actual role of apoE4 in the development of AD is yet unclear, the knowledge of the pathological alterations in other isoforms during AD development may lead to a promising breakthrough in AD pathology.

The cysteine–arginine interchange at 112, 158, or both is one of the major features that distinguish apoE2 and apoE3 from apoE4. The presence of cysteine allows apoE2 and apoE3 to form homodimers through a disulfide bond [6]. These isoforms also bind to apoAII, a plasma protein of 8.7 kDa, via their cysteine residue to form apoE heterodimers, i.e., apoE-AII complex (apoE-AII) and apoAII-E2-AII complex [7,8]. Previous studies have shown that these variations impact the distribution of apoE in lipoprotein particles; the preference of apoE3 for high-density lipoprotein (HDL) and apoE4 for very low-density lipoprotein (VLDL) results from the differences in the charge at residue 112 and the ability to form apoE-AII [9]. These characteristics also affect the pathophysiological functions of apoE. Several studies have demonstrated that the dimerization of apoE3 results in a significant increase in the synthesis of HDL [10,11].

Cysteine thiol, a highly reactive residue in proteins, is subjected to post-translational redox-mediated modifications (e.g., hyperoxidation, nitrosylation, glutathionylation, and palmitoylation) [12,13]. To date, several studies have suggested that the redox status of cysteine thiol may greatly affect various biological activities [14–16]. We have recently suggested that the redox status of serum apoE may depend on its cysteine thiol modification and is closely related to the synthesis of HDL [17].

Redox-mediated alterations of cysteine thiol residues are either reversible or irreversible, and apoE disulfide complexes such as homodimers and heterodimers correspond to the forms with reversible alterations. The irreversible modification of the cysteine thiol residue is predicted to cause loss-of-function, leading to the degradation of the modified protein [18,19]. In contrast, the temporary alteration of a protein due to reversible modifications is crucial for the protection of the protein from irreversible detrimental changes as well as for the modulation of protein function [20]. Based on their reversible properties, the apoE disulfide-linked complexes may be committed to maintaining their redox status and regulating their functions, consequently impacting the pathophysiology of various apoE-related diseases such as AD and cardiovascular diseases.

Based on the above hypothesis, we investigated the effect of apoE-AII on oxidative stress in subjects with apoE3, the most common isoform, to clarify its role in the redox status of apoE-containing lipoproteins.

## Materials and methods

### Materials

Photocleavable maleimide-conjugated polyethylene glycol (PEG-PC-Mal; MW, 2736 Da) was purchased from Dojindo Molecular Technologies, Inc. (Kumamoto, Japan). Recombinant apoE3 was obtained from BioVision Inc. (Milpitas, CA, U.S.A.) and horseradish peroxidase (HRP)-conjugated anti-apoE polyclonal antibody was supplied by Academy Bio-medical Co. (Houston, TX, U.S.A.). All other chemicals used were of the highest grade.

### Preparation of apoE3-containing lipoprotein fractions

Serum apoE3-containing lipoproteins, VLDL (<1.006 kg/l) and HDL (1.063–1.21 kg/l), were isolated from the sera of healthy volunteers with apoE phenotype E3/E3 (*n*=3, apoE, 27.0 ± 6.1 mg/l) using an ultracentrifugation method as previously described [21]. The serum apoE phenotype was determined by isoelectric focusing and immunoblot analysis as previously described [22]. The present study was approved by the Tsukuba University Ethics Committee and all volunteers provided written informed consent.

### Analysis of apoE redox status

**Figure 1:**
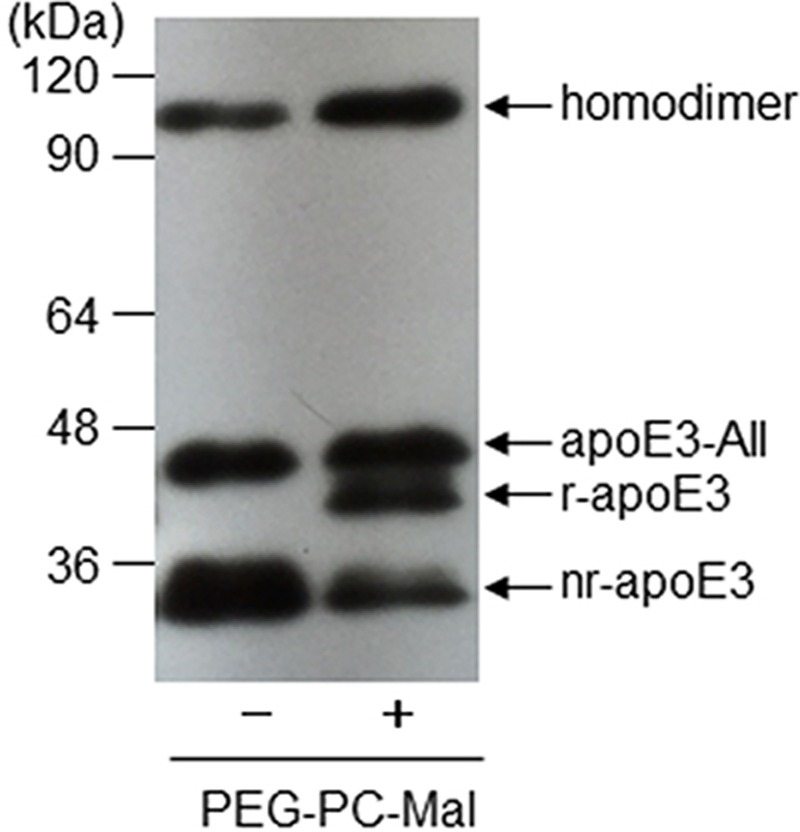
Typical pattern of the band-shift assay using polyethylene glycol maleimide (PEG-PC-Mal) Healthy volunteer’s serum was incubated with PEG-PC-Mal. The band-shift assay was performed using HRP-conjugated anti-apoE antibody, as described in Materials & Methods.

The redox status of apoE was analyzed with a band-shift assay using polyethylene glycol maleimide (PEG-PC-Mal), according to our previous study [17]. Briefly, PEG-PC-Mal was added to the sample at a final concentration of 1.0 mmol/l. The mixture was incubated for 30 min at 37°C, followed by electrophoresis using 10% SDS/PAGE under non-reducing condition. After electrophoresis, the gel was UV-irradiated for 15 min to eliminate the PEG moiety, and the separated proteins were transferred onto a polyvinylidene fluoride membrane. The blots were probed with an HRP-conjugated anti-apoE polyclonal antibody. The specific bands were developed using an ECL detection kit (Nacalai Tesque, Inc., Kyoto, Japan), and were analyzed using ImageJ 1.45 software from the National Institutes of Health. As previously described [17], a 40-kDa labeled apoE3 was defined as the reduced form (r) of apoE3, while the monomeric form (35-kDa unlabeled apoE3) remaining in the presence of PEG-PC-Mal was termed as the non-reduced form (nr) of apoE3 (Figure 1). In addition to these bands, as previous studies have well confirmed [6,9], the bands with molecular masses of 47.0 and 95.0 kDa were defined as the apoE3-AII and homodimer, respectively. The levels of the homodimer, apoE3-AII, reduced-form apoE (r-apoE3), and nr-apoE3 were estimated from the densitometric values of these bands.

### Determination of apoE and apoAII

The levels of apoE and apoAII were determined by enzyme-linked immunosorbent assay and turbidimetric immunoassay, respectively, using a commercially available kit.

### Statistical methods

Data are presented as mean ± standard error (S.E.). Statistical analysis was performed using either a paired *t*-test or an unpaired *t*-test depending on the dataset. A value of *P<*0.05 was considered as significant.

## Results

### Redox profiles of apoE3 in VLDL and HDL

The redox profiles of apoE3 in VLDLs and HDLs isolated from the sera of three healthy volunteers with apoE3/E3 phenotype were determined by the band-shift assay using PEG-PC-Mal. As per our previous findings [17], naive VLDL and HDL showed differences in the redox status of apoE3; i.e., r-apoE3 was more dominant in VLDL than in HDL (*P*<0.05), whereas the apoE3-AII level was higher in HDL (*P*<0.05) (Table 1).

**Table 1:** Redox Profiles of apoE3 in VLDL and HDL

Sample	apoE (mg/l)	fraction (%)			
		r-apoE3	nr-apoE3	apoE3-AII	homodimer
VLDL					
S1	15.1	15.2	27.5	39.6	17.8
S2	12.6	17.9	29.3	33.1	19.7
S3	14.2	21.9	25.7	34.3	18.1
mean ± SE	14.0 ± 0.7*	18.33 ± 1.95*	27.50 ± 1.04	35.67 ± 2.00*	18.53 ± 0.59
HDL					
S1	10.4	11.5	30.6	42.0	16.0
S2	11.0	7.7	25.8	45.1	21.4
S3	12.1	10.4	28.3	44.4	16.9
mean ± SE	11.2 ± 0.5	9.87 ± 1.13	28.23 ± 1.39	43.83 ± 0.94	18.10 ± 1.67

S1∼S3, sera obtained from healthy volunteers with apoE phenotype E3/E3.

* *P*<0.05 (vs HDL).

### Influence of oxidative stress on the redox status of apoE3

**Figure 2:**
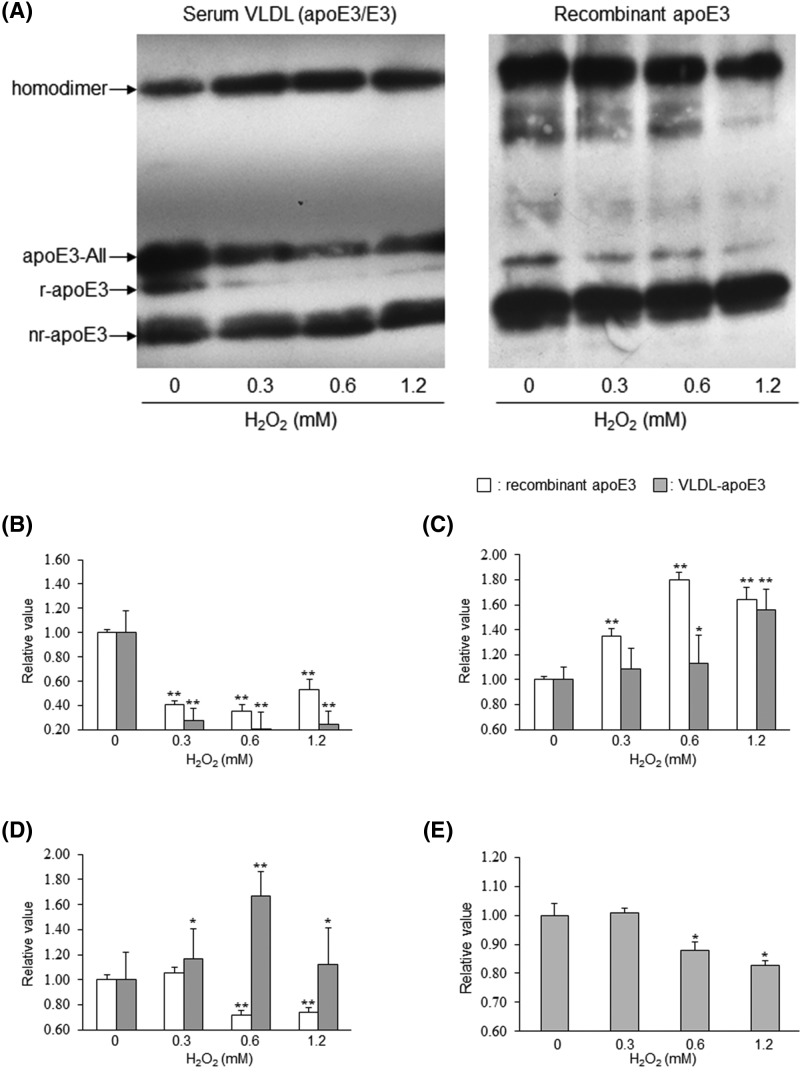
Influence of H_2_O_2_ oxidation on the redox status of apoE3 Recombinant apoE3 (10.0 mg/l) or each of the three VLDLs (*n*=3, apoE concentration, 12.0 ± 2.9 mg/l) was incubated with serially diluted H_2_O_2_ at final concentrations of 0, 0.3, 0.6, and 1.2 mM for 15 min at 37°C. The reaction was quenched by the addition of tris (2-carboxyethyl) phosphine into ammonium bicarbonate buffer, and the band-shift assay was carried out using PEG-PC-Mal as described in Materials & Methods (**A**). After densitometry, we analyzed the changes in the relative levels of r-apoE3 (**B**), nr-apoE3 (**C**), homodimer (**D**), and apoE3-AII (**E**) and compared the values with those obtained for the non-oxidized controls. The values shown are the mean ± S.E. from duplicate measurements of each sample from three independent experiments. *, *P<0.05* and **, *P<0.001* as compared with the non-oxidized recombinant apoE3 or VLDL (0 mM H_2_O_2_).

To investigate the influence of oxidative stress on the redox status of apoE3, we added hydrogen peroxide (H_2_O_2_) at final concentrations of 0, 0.3, 0.6, and 1.2 mM to either recombinant apoE3 or to each of the three above-mentioned VLDLs. We performed the band-shift assay using these mixtures (Figure 2A). The oxidation with H_2_O_2_ significantly decreased the levels of r-apoE3 in both recombinant apoE3 (0.43-fold, *P<*0.001) and VLDL (0.24-fold, *P<*0.001) compared with the non-oxidized controls (Figure 2B). In contrast, the oxidation with H_2_O_2_ dose-dependently increased nr-apoE3 levels in both recombinant apoE3 and VLDL (Figure 2C). However, a gradual change in nr-apoE3 level was observed in VLDL compared with in recombinant apoE3. The fluctuations in homodimer levels were different between recombinant apoE3 and VLDL (Figure 2D). The homodimer levels in recombinant apoE3 were significantly reduced upon oxidation at more than 0.6 mM H_2_O_2_ concentration (0.73-fold, *P<*0.001), although the level was almost unchanged following oxidation with 0.3 mM H_2_O_2_. In contrast, the homodimer levels in VLDL were significantly increased following oxidation and displayed a peak value upon incubation with 0.6 mM H_2_O_2_ (1.67-fold, *P<*0.001). The levels of apoE3-AII present in VLDL, but not in recombinant apoE3, significantly decreased following oxidation with more than 0.6 mM H_2_O_2_ (*P<*0.05), although no change was observed in response to treatment with 0.3 mM H_2_O_2_ (Figure 2E).

### Influence of apoAII on the redox status of apoE3

**Figure 3:**
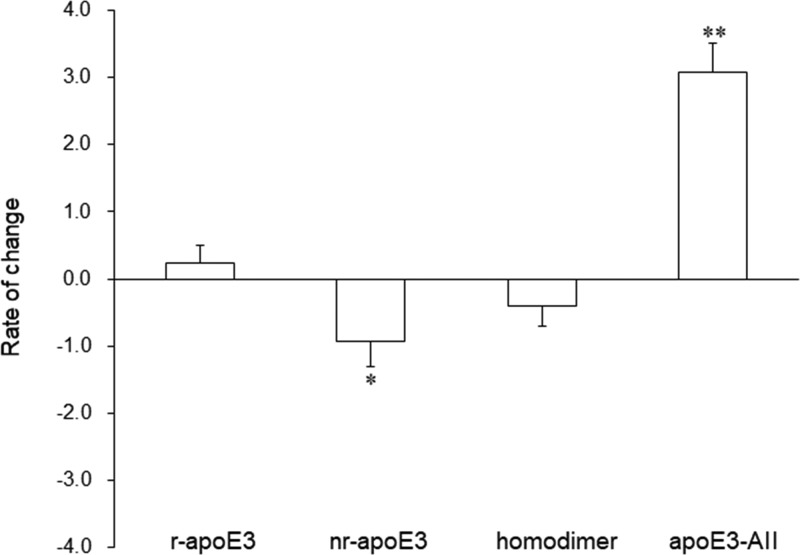
Influence of apoAII on the redox status of apoE3 Each of the three VLDLs was incubated with an equal volume of saline or recombinant apoAII at a final concentration of 650 mg/l at 37°C overnight. The band-shift assay using these mixtures was performed as described in Materials & Methods. After densitometry, we analyzed the rate of change in r-apoE3, nr-apoE3, homodimer, and apoE3-AII levels. The values shown are the mean ± S.E. from duplicate determinations of each sample from three independent experiments. The values shown are the mean ± S.E. from duplicate determinations of each sample from three independent experiments. *, *P<0.005 and* **, *P<0.001*.

To assess the influence of apoAII on the redox status of apoE3 in naïve VLDL, we incubated each of the three above-mentioned VLDLs with an excess of recombinant apoAII at a final concentration of 650 mg/l and performed the band-shift assay. The formation of apoE3-AII was significantly induced after the addition of the recombinant apoAII (*P<*0.001), and a less than 5% reduction in nr-apoE3 level was observed (*P<*0.005). No differences in the levels of r-apoE3 and the homodimer were observed in the presence and absence of recombinant apoAII (Figure 3).

### Effect of apoAII against oxidative stress on apoE3

**Figure 4:**
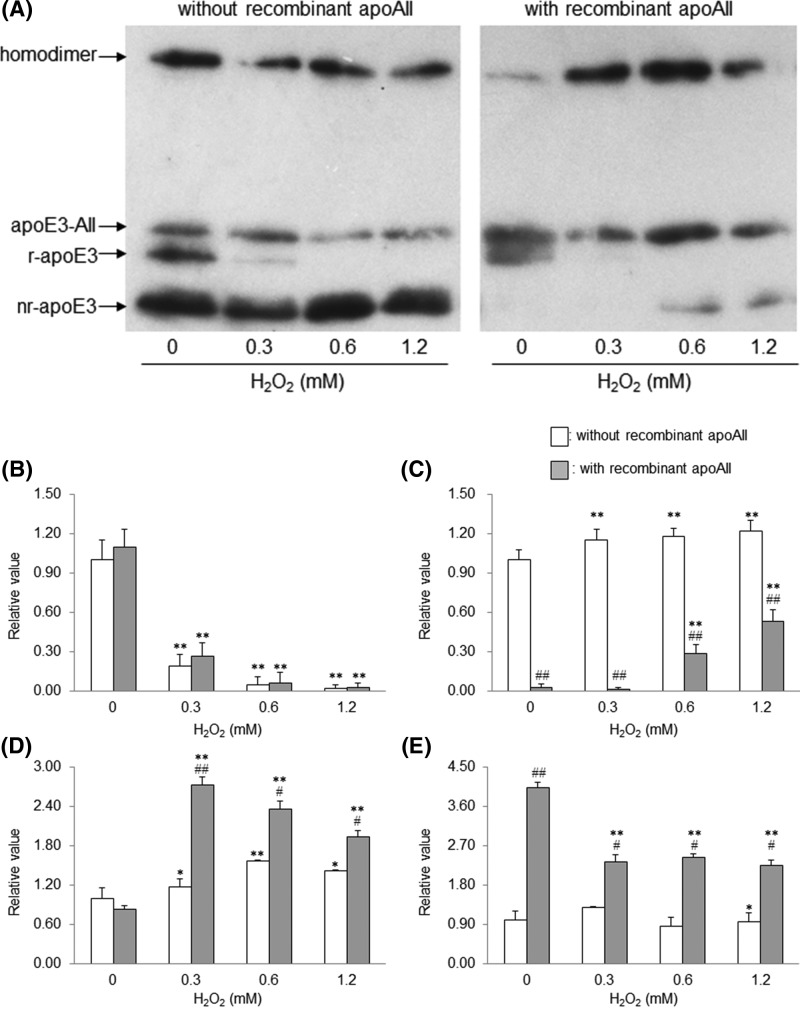
Effect of apoAII on the oxidative stress against apoE3 Each of the three VLDLs was incubated with an equal volume of saline or recombinant apoAII at a final concentration of 650 mg/l at 37°C overnight, followed by oxidation with H_2_O_2_ at a final concentration of 0, 0.3, 0.6, or 1.2 mM for 15 min at 37°C. The reaction was quenched by the addition of tris (2-carboxyethyl) phosphine in ammonium bicarbonate buffer, and the band-shift assay was carried out using PEG-PC-Mal, as described in Materials & Methods (**A**). The band intensities of r-apoE (**B**), nr-apoE (**C**), homodimer (**D**), and apoE3-AII (**E**) were determined through densitometry. The values shown are the mean ± S.E. from duplicate determinations of each sample from three independent experiments. *, *P<0.05* and **, *P<0.001* as compared with each non-oxidized control (0 mM H_2_O_2_). ^#^, *P<0.01* and ^##^, *P<0.001* as compared with each recombinant apoAII-free sample under the same oxidation conditions.

We examined whether apoAII acts against oxidative stress on apoE3 and evaluated its role in the maintenance of redox status. We incubated each of the three above-mentioned VLDLs with recombinant apoAII and subjected the samples to oxidation with H_2_O_2_. The band-shift assay was performed using these mixtures (Figure 4A). Regardless of the presence or absence of the recombinant apoAII, r-apoE3 levels reduced in response to H_2_O_2_-mediated oxidation (Figure 4B). On the other hand, nr-apoE3 levels showed a dose-dependent increase in the absence of recombinant apoAII following treatment with H_2_O_2_. In the presence of recombinant apoAII, although nr-apoE3 levels were significantly induced upon oxidation with more than 0.6 mM H_2_O_2_, the levels were lower than those in the absence of recombinant apoAII. In addition, similar to the non-oxidized control, nr-apoE3 was almost undetectable under oxidizing conditions with 0.3 mM H_2_O_2_ (Figure 4C). Homodimer levels increased following H_2_O_2_-mediated oxidation, irrespective of the presence or absence of recombinant apoAII; however, different fluctuation patterns were observed between the two conditions. Homodimer levels increased significantly more in the presence rather than in the absence of recombinant apoAII (2.61-fold for the oxidizing condition with 0.3 mM H_2_O_2_, *P*<0.001). In addition, homodimer levels peaked in the presence of recombinant apoAII following oxidation with 0.3 mM H_2_O_2_. On the contrary, in the absence of recombinant apoAII, the peak value was observed following oxidation with 0.6 mM H_2_O_2_. Under both conditions, the H_2_O_2_ concentration required to attain the peak value of homodimer levels was equal to that required to obtain the lowest level of apoE3-AII (Figure 4D). The apoE3-AII levels decreased by approximately 60% in comparison to the levels observed for the non-oxidized control group in the presence of more than 0.3 mM H_2_O_2_; however, the levels remained almost constant regardless of H_2_O_2_ concentration and were significantly higher than those in the absence of recombinant apoAII (1.78–2.78-fold, *P*<0.01) (Figure 4E).

### Influence of oxidation on the redox status of apoE3-containing lipoproteins

**Figure 5:**
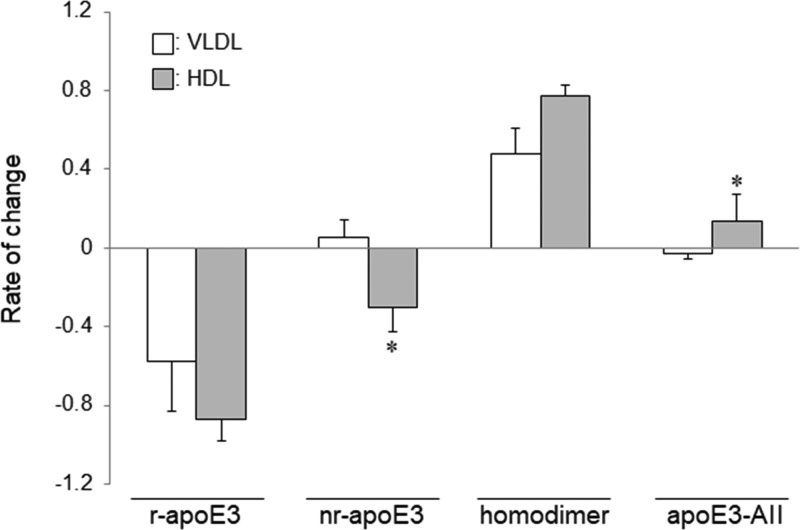
Influence of oxidation on the redox status of apoE3-containing lipoproteins Each of the three VLDLs and HDLs was adjusted to equal the apoE concentration and incubated with 0.6 mM H_2_O_2_ for 15 min at 37°C. The reaction was quenched by the addition of tris (2-carboxyethyl) phosphine in ammonium bicarbonate buffer, and the band-shift assay was carried out using PEG-PC-Mal as described in Materials & Methods. After densitometry, we analyzed the rate of change in r-apoE3, nr-apoE3, homodimer, and apoE3-AII levels. The values shown are the mean ± S.E. from duplicate determinations of each sample from three independent experiments. *, *P<0.005*.

To examine whether the behavior of the apoE3 redox status under oxidative stress was different among lipoproteins, we incubated each of the three above-mentioned VLDLs and HDLs with 0.6 mM H_2_O_2_ and performed the band-shift assay using these mixtures. As previously demonstrated [23], apoAII was more abundant in HDL (677 ± 93 mg/l) than in VLDL (undetectable). In accordance with the above results, H_2_O_2_ oxidation decreased the level of r-apoE3 and increased the level of homodimer. The rate of change in r-apoE3 and homodimer levels from HDL tended to be greater than that from VLDL. On the other hand, the alterations in nr-apoE3 and apoE3-AII levels from HDL were different from those from VLDL. Under oxidative stress, HDL showed a significant decrease, rather than an increase, in the nr-apoE3 level with a concomitant significant increase in the apoE3-AII level (*P*<0.005), whereas VLDL showed no significant changes (Figure 5).

## Discussion

Oxidative stress causes reversible or irreversible protein modifications and is involved in the pathogenesis of various diseases [24–26]. The reversible modification of the cysteine thiol of proteins, represented by disulfide bond formation, has a significant impact on diverse physiological functions by protecting proteins from irreversible detrimental changes [20]. Hence, the present study was designed to test whether the disulfide-linked complexes of apoE3, especially apoE3-AII, play a protective role against oxidative stress.

The redox profiles of apoE3 in VLDL and HDL were consistent with those in our previous studies [17], although these data derived from small-scale experiments. Further studies will be necessary to clarify whether the intensities of the apoE bands developed using HRP-conjugated anti-apoE polyclonal antibody are comparable among r-apoE, nr-apoE, apoE3-AII, and the apoE homodimer. However, relative but not absolute comparison may be sufficient, even by the present method.

We showed the differences in the behavior of recombinant apoE3 and apoE3 from VLDL to oxidative stress. The oxidative stress–induced increase in nr-apoE3 was more prominent in recombinant apoE3 than in VLDL, suggesting that recombinant apoE3 is more susceptible to oxidative stress as compared with apoE3 from VLDL. In the case of recombinant apoE3, the reduction in the levels of r-apoE3 and the homodimer was obviously related to the increase in the level of nr-apoE. Hence, the oxidation state of nr-apoE3 may be higher than that of the homodimer. In other words, further oxidation of nr-apoE3 may be more difficult to achieve in comparison with that of the homodimer. It is interesting that the increase in the level of nr-apoE3 in VLDL was not as remarkable as that observed in recombinant apoE3. In addition, a marked increase in the level of homodimers was observed for VLDL rather than for recombinant apoE3, consistent with the reduction in the level of apoE3-AII. These results suggest that the difference in the fluctuation of homodimer levels between recombinant protein and VLDL may be attributed to the presence or absence of apoE3-AII. The newly synthesized homodimer in VLDL after H_2_O_2_-mediated oxidation may comprise apoE3 molecules derived from apoE3-AII; i.e., the redox reactivity of the homodimer is lower than that of apoE3-AII.

Also of interest are the notable effects of apoAII on the redox status of apoE3 in naive VLDL. The addition of an excessive amount of recombinant apoAII dramatically reduced the level of nr-apoE3 with a simultaneous formation of apoE3-AII, suggesting that the majority of nr-apoE3 (PEG-PC-Mal-unlabeled apoE3) may be present in a reversible oxidized form, derived from modifications, such as sulfenylation, S-nitrosylation, S-glutathiolation, and S-cysteinylation, even though we have previously postulated that nr-apoE3 was an irreversible oxidized form [17]. On the other hand, there is no doubt that an oxidation-induced nr-apoE3 is the true irreversibly oxidized form.

Intriguingly, the presence of excessive apoAII prominently suppressed the increase in nr-apoE3 levels with a concomitant significant increase in the level of apoE3-AII and caused a greater increase in homodimer formation under lower oxidative stress. Also, the increase in the level of homodimers seemed associated with a simultaneous reduction in the level of apoE3-AII. Overall, these findings suggest that apoE3-AII and the homodimer may possibly be involved in imparting protection from oxidative stress while maintaining equilibrium with each other.

It is well known that apoAII exists predominantly in the HDL fraction [23]. The results of the present study are in line with those previously reported. In addition, previous studies have demonstrated that apoE3-AII and the homodimer prefer HDL over VLDL [9]. On the basis of these evidences, it is expected that apoE3 in HDL would be more resistant to oxidation than apoE3 in VLDL. Indeed, nr-apoE3 levels in HDL decreased after oxidation, in contrast with the result observed with VLDL. This finding suggests that most of nr-apoE3 in HDL may exist as a reversible oxidized form and may protect apoE3 molecules from further oxidation through the formation of apoE3-AII or the homodimer. On the contrary, the increased nr-apoE3 in VLDL may be the irreversibly oxidized form derived from r-apoE3 and disulfide-linked complexes. The opposite changes in apoE3-AII between HDL and VLDL may be associated with the significant differences in the amount of apoAII. As HDL is rich in apoAII, the substrate for apoE3-AII, the formation of the complex is facilitated in response to oxidative stress and may consequently prevent the further oxidation of the cysteine thiol of apoE3. By contrast, apoE3-AII in VLDL, which originally contains a low concentration of apoAII, may be susceptible to further oxidation. Of note, the rate of change of the apoE homodimer level tended to be higher for HDL than in VLDL, suggesting that the homodimer may also serve as a buffer against irreversible oxidation by maintaining an equilibrium with apoE3-AII. In addition, it would be interesting if the dimerization of apoE3 was also found to protect against the oxidation of other highly reactive amino acid residues, such as methionine.

We hypothesize that although oxidative stress causes a graded increase in the oxidation state of the cysteine thiol in the apoE3 molecule, the equilibrium between apoE3-AII and the homodimer can prevent the irreversible oxidation, especially under apoAII- or apoE3-AII–abundant conditions. Disulfide bond formation may maintain the function of the apoE3 molecule, although the redox reactivities of apoE3-AII and the homodimer are lower than that of monomeric r-apoE3. This hypothesis is strongly supported by the fact that apoE3 shows a high ability to synthesize HDL, which is attributable to the formation of disulfide-linked complexes [9]. However, one limitation of our study was the use of H_2_O_2_ at high concentrations, which are far from physiological. Hence, further experiments under near physiological conditions will be necessary to confirm the pathophysiological effects of oxidative stress.

As apoE4 with various detrimental properties [3,27] exists as the only monomeric form owing to the absence of any cysteine residue, the monomeric irreversibly oxidized apoE3 may behave like apoE4. Although further studies are warranted to examine how the redox status of apoE3 affects its function, our observations in the present study suggest that the formation of the disulfide-linked complexes is certainly beneficial for maintaining the redox status of apoE3 by preventing the formation of the detrimental irreversibly oxidized form in response to oxidative stress. The disulfide-linked complexes of apoE3, especially apoE3-AII, may be implicated in the pathophysiology of apoE-related diseases such as AD.

## Abbreviations

AD: Alzheimer’s disease
apo: apolipoprotein
apoE-AII: apoE-AII complex
HDL: high-density lipoprotein
HRP: horseradish peroxidase
Mal: maleimide
nr-apoE: non-reduced-form apoE
PC: photocleavable
PEG: polyethylene glycol
r-apoE: reduced-form apoE
VLDL: very low-density lipoprotein
